# A Narrative Review of Advances in Skin Tissue Engineering: From Physiological Architecture to 3D Bioprinting

**DOI:** 10.7759/cureus.113585

**Published:** 2026-07-29

**Authors:** Swasti Bhatnagar, Deepsekhar Das

**Affiliations:** 1 Ophthalmology, All India Institute of Medical Sciences, New Delhi, IND

**Keywords:** 3d bioprinting, bioinks, eggshell membrane (esm), extracellular matrix, regenerative medicine, skin tissue engineering, wound healing

## Abstract

The skin is a complex, multilayered organ. Damage from burns, trauma, chronic ulcers, or surgical defects often outstrips the regenerative capacity of conventional healing. Traditional skin grafting, while standard, is limited due to donor site morbidity, lack of appendages, and altered biomechanical/sensory properties. This narrative review traces the evolution of skin repair from autografting to bioengineered substitutes and 3D bioprinting, emphasizing physiological design principles, biomaterials, and translational challenges. The literature synthesis mainly focused on skin architecture and wound-healing phases, limitations of epidermal/dermal grafts, development of acellular and cellular artificial skin, recent advances in bioinks, extrusion/inkjet/laser-assisted bioprinting, and functional appendage regeneration. Ophthalmic applications, including corneal and eyelid reconstruction, were also reviewed. Artificial skin substitutes evolved from acellular scaffolds to cell-seeded constructs using keratinocytes, fibroblasts, and decellularized extracellular matrix (ECM). Key biomaterials include hydrogels, collagen, and gellan gum as well as novel composites like eggshell membrane-alginate bioinks. In vivo models demonstrate accelerated re-epithelialization, organized collagen and vascularization with bioprinted grafts. Emerging technologies, such as 4D bioprinting with shape-memory polymers, smart dressings with biosensors, and in situ robotic bioprinting, promise adaptive, real-time wound repair. Biomaterials and fabrication strategies developed for cutaneous repair are increasingly relevant to ophthalmology and broader regenerative medicine. 3D bioprinting provides a viable translational route to functional, patient-specific artificial skin, overcoming key graft limitations. Future work must advance vascularized, multilayer constructs and move toward standardized, regulatory-aligned fabrication for clinical translation.

## Introduction and background

**Figure 1:**
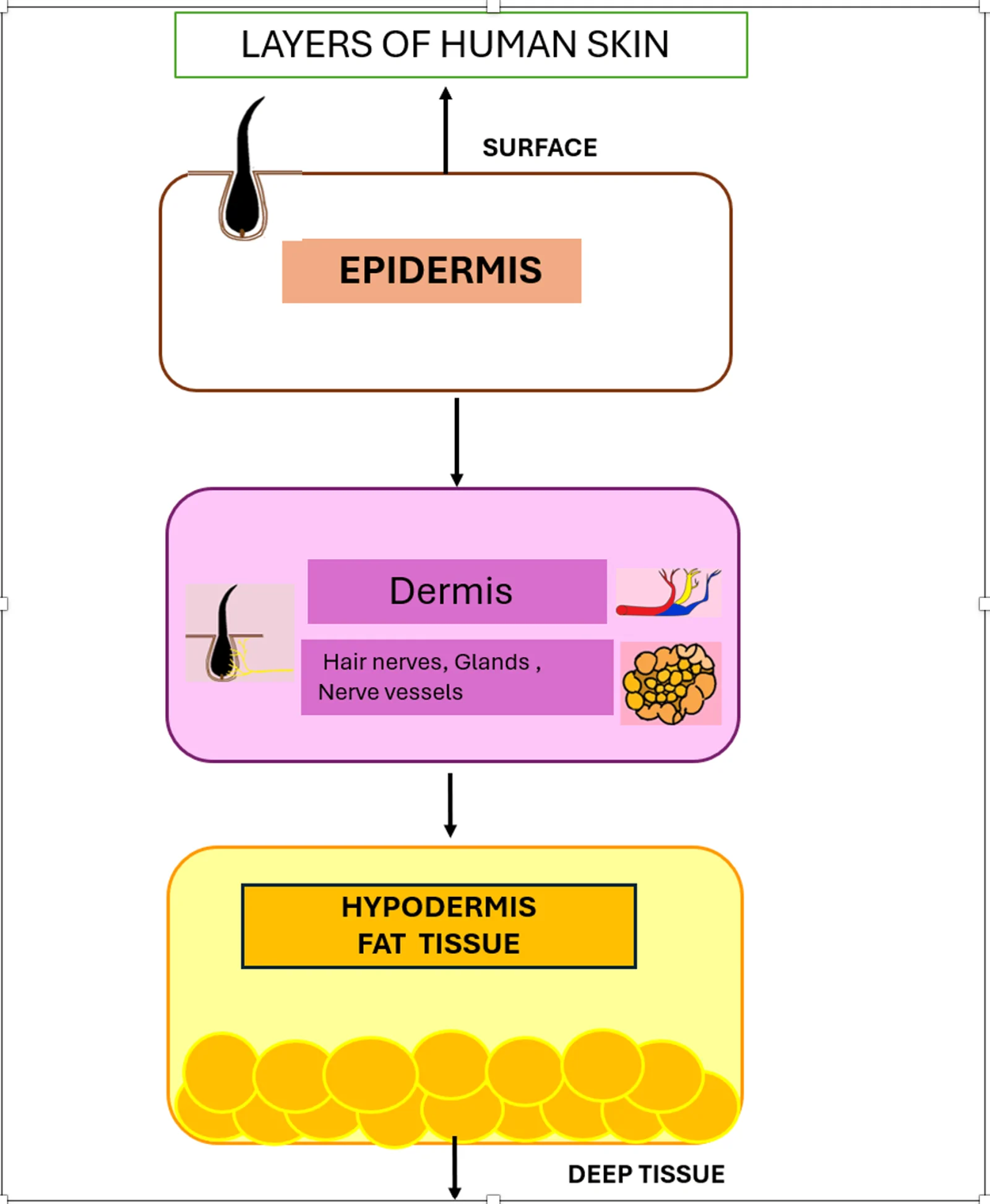
Schematic representation of human skin illustrating the epidermis, dermis, and hypodermis and their major anatomical components Schematic representation of human skin illustrating the epidermis (barrier), dermis (hair follicles, glands, nerves, vessels), and hypodermis (fat for insulation and cushioning) and their major anatomical components. This structural overview supports skin tissue engineering and the design of artificial skin. Image created by the authors using Microsoft PowerPoint (Microsoft Corporation, Redmond, WA, US).

The skin of human beings can be considered a complex organ system due to the presence of functionally arranged cell types in three distinct layers (Figure 1) [1].

The epidermis acts as an external layer of protection for the integumentary system and consists primarily of keratinocytes [2]. Beneath the epidermis lies the basement membrane, followed by the dermis, which mainly consists of fibroblasts that produce an abundant extracellular matrix (ECM) containing collagen and elastin [3]. The deepest layer is known as the hypodermis, which is primarily composed of adipocytes [4]. These cells serve as an important energy reservoir for storage purposes, while also providing shock absorption and secreting various bioactive compounds [5].

Since each layer of the skin plays distinct structural and biological roles, damage to these layers triggers a complex and coordinated wound healing response. Skin repair begins with hemostasis, where the rapid action of platelets and the clotting mechanism form an initial fibrin clot to close the wound. The inflammatory phase involves the migration of immune cells to the site of injury, where they clear dead tissues and pathogens while facilitating tissue repair. The proliferative phase includes angiogenesis, fibroblast activity, extracellular matrix deposition, and re-epithelialization of the wounded area.

The remodelling phase strengthens the healing tissue through collagen reorganization and wound contraction. Several factors can impair the wound healing process, resulting in chronic injuries or excessive scarring (Table 1) [6].

**Table 1:** Factors affecting wound healing and their mechanisms of action

Reference	Factor	Explanation
Gonzalez et al. [6]	Wound pH	An alkaline pH promotes bacterial growth, whereas a slightly acidic pH supports healing.
Sachdeo et al. [7].	Comorbidities (e.g., Diabetes, Vascular Issues)	Underlying conditions, such as diabetes and vascular disorders, impair healing through poor circulation, neuropathy, and metabolic abnormalities.
Przekora et al. [8]	Excessive Proteases (MMPs) and Oxidative Stress: Fibroblast and ECM Dysregulation	Elevated MMPs and ROS damage the extracellular matrix and surrounding tissues, impairing repair. Abnormal fibroblast activity and excessive ECM deposition can lead to delayed healing or severe scarring.
Valencia et al. [9]	Patient Characteristics and Lifestyle	Age, nutritional status, medications, smoking, and patient compliance influence healing outcomes.
Mahdavi et al. [10]	Wound Hydration and Exudate Management	Optimal moisture balance promotes cell migration, re-epithelialization, and efficient wound healing.

Chronic wounds occur in an estimated 6.5 million people each year in the USA, costing more than $25 billion per annum to treat [11]. In total, burns account for an estimated 180,000 fatalities worldwide each year, and many others survive with considerable impairments. While developments have been made in wound therapy, traditional methods are still inadequate for large-scale wounds [12].

The natural wound-healing process may be inadequate in cases of extensive damage that include severe burns or major trauma, frequently leading to delayed healing, chronic non-healing wounds, or excessive scarring. Critically, while the wound-healing cascade is well-characterized, the translation of this knowledge into effective therapeutic interventions remains limited, highlighting a persistent gap between mechanistic understanding and clinical application.

Although several reviews have addressed skin tissue engineering and bioprinting individually, few have systematically traced the translational pathway from conventional grafting through advanced biofabrication while specifically highlighting the unique contributions of emerging biomaterials such as eggshell membrane. Furthermore, the cross-disciplinary applicability of these technologies to ophthalmology remains underexplored.

This narrative review aims to: (i) trace the evolution of skin repair from conventional auto grafting to bioengineered substitutes and 3D bioprinting, (ii) evaluate the physiological design principles, biomaterials, and bio-ink formulations that underpin current tissue engineering strategies,(iii) highlight the translational potential of these technologies, and (iv) identify key limitations, knowledge gaps, and future directions for clinical translation. Having established the clinical need for advanced skin repair strategies, the following section describes the methodology used to conduct this literature review.

## Review

Methodology

A thorough literature search was carried out using the PubMed, Scopus, and Web of Science databases using the primary search terms “3D bioprinting”, “artificial skin”, “skin graft”, “bioink”, “eggshell membrane”, and “bioprinting”. A total of 766 studies were found. Of these, 368 duplicate studies were removed, leaving behind 398 unique studies. Out of the 398 unique studies, 309 studies were found to be irrelevant to the purpose of this review. The remaining 89 studies were further reviewed for their eligibility. Based on the review, out of these, 51 studies were found to be irrelevant to the scope of this review and were removed. Thus, 38 studies were found to be relevant.

Skin grafting: principles, clinical applications, limitations, and emerging advances

Skin grafting surgery is a surgical process that involves the transfer of skin, and sometimes its adnexal structures, from one area of the body to another. Unlike skin flaps, a free skin graft is completely detached from its original vascular supply and relies entirely on the formation of new vessels from the recipient site for survival and integration [13].

Skin grafts are primarily categorized according to the thickness of donor tissues such as epidermal skin grafts (ESGs), split-thickness skin grafts (STSGs), and full-thickness skin grafts (FTSGs) (Table 2) [8].

**Table 2:** Classification of skin grafts based on donor tissue thickness

Graft Type	Composition	Characteristics and Use
Epidermal Skin Grafts (ESGs)	Contains only the epidermal layer of the skin.	Typically used for superficial or small wounds when complete restoration of skin function is not required.
Split-Thickness Skin Grafts (STSGs)	Consists of the epidermis and a variable portion of the dermis.	Further subdivided into thin (0.125–0.275 mm), medium (0.275–0.4 mm), and thick (0.4–0.75 mm) grafts.
Full-Thickness Skin Grafts (FTSGs)	Includes the epidermis and the entire dermis.	Includes all dermal components and sometimes various amounts of subcutaneous tissue; used when aesthetic outcome is essential.
Composite Grafts	Composed of at least two different tissue types.	Most commonly composed of skin and cartilage; particularly useful for repairing defects such as the nasal alar rim.

Skin grafting is a widely used reconstructive technique with several important clinical indications, including the treatment of deep partial-thickness and full-thickness burns, chronic ulcers (such as venous leg ulcers, pressure ulcers, and diabetic foot ulcers), and the closure of post-surgical defects following the excision of cutaneous malignancies or large congenital birthmarks. Despite its clinical utility, skin grafting has several physiological and functional limitations. Most conventional skin grafts fail to regenerate skin appendages such as hair follicles and sweat glands. Furthermore, grafted skin is often less flexible and more brittle than native skin because of alterations in collagen architecture, including reduced collagen fiber thickness, and sensory function is frequently diminished following grafting [9].

Following transplantation, the skin graft undergoes a series of well-coordinated biological events that are essential for graft survival and integration. These include revascularization, during which endothelial cells and pericytes within the graft self-assemble into microvascular networks that subsequently inosculate with the host's angiogenic vessels at the wound site [14]. Recent advances in three-dimensional bioprinting have enabled the fabrication of skin grafts capable of regenerating complex skin appendages in vivo. Skin-derived precursors (SKPs) and dermal papilla cells interact to promote the formation of hair follicle units, resulting in new hair growth within the grafted tissue [15].

Moreover, the extracellular matrix of bioprinted grafts undergoes dynamic remodelling; organized collagen arrangement reduces wound contraction, minimizes hypertrophic scar formation, and improves the tensile strength of the regenerated tissue [16]. Given the limitations of conventional skin grafting, researchers have turned to tissue engineering approaches to develop artificial skin substitutes capable of overcoming these challenges.

Artificial skin: from skin grafting to tissue engineering

**Figure 2:**
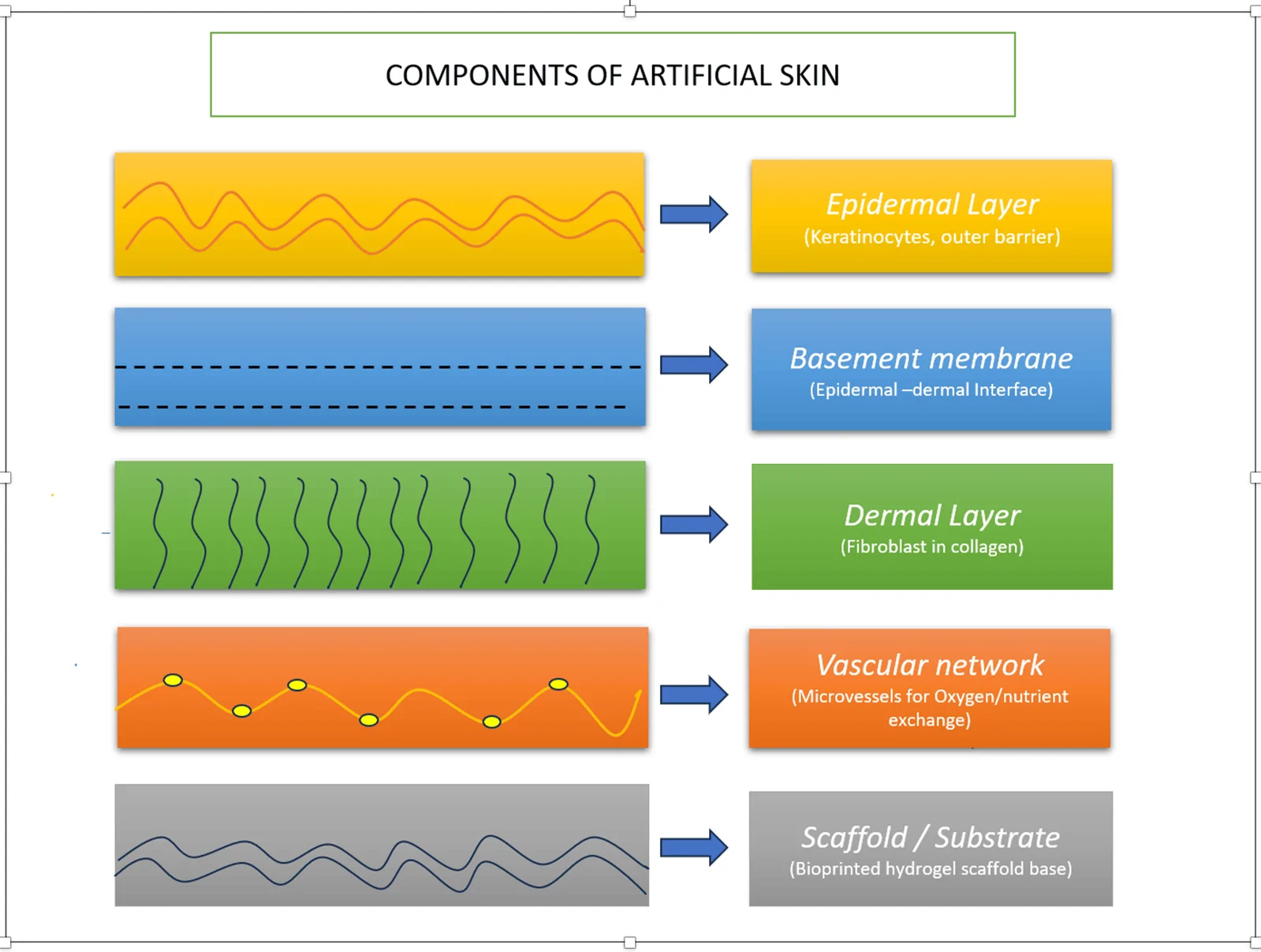
Major functional layers and components of artificial skin Major functional layers and components of an artificial skin. The key layers include (i) an epidermal layer composed of keratinocytes forming an outer protective barrier, (ii) a basement membrane serving as an interface between the epidermal and dermal layers and supporting cell adhesion, (iii) a dermal layer consisting of fibroblasts embedded within a collagen-based extracellular matrix, providing structural and mechanical support, (iv) a vascular network of microvessels enabling oxygen and nutrient exchange within the construct, and (v) a scaffold/substrate layer, typically a bioprinted hydrogel, serving as the structural base supporting the overlying layers. Image created by the authors using Microsoft PowerPoint (Microsoft Corporation, Redmond, WA, US).

Artificial skin refers to a bioengineered skin substitute intended to provide replacement and regenerative capacity for skin tissues affected by burns, trauma, wounds, and surgical defects [8]. The substitute works like natural skin for cell attachment and proliferation [9]. It usually comprises living cells (keratinocytes, fibroblasts, biomaterials, extracellular matrix (ECM), and bioactive substances used in tissue regeneration (Figure 2).

**Figure 3:**
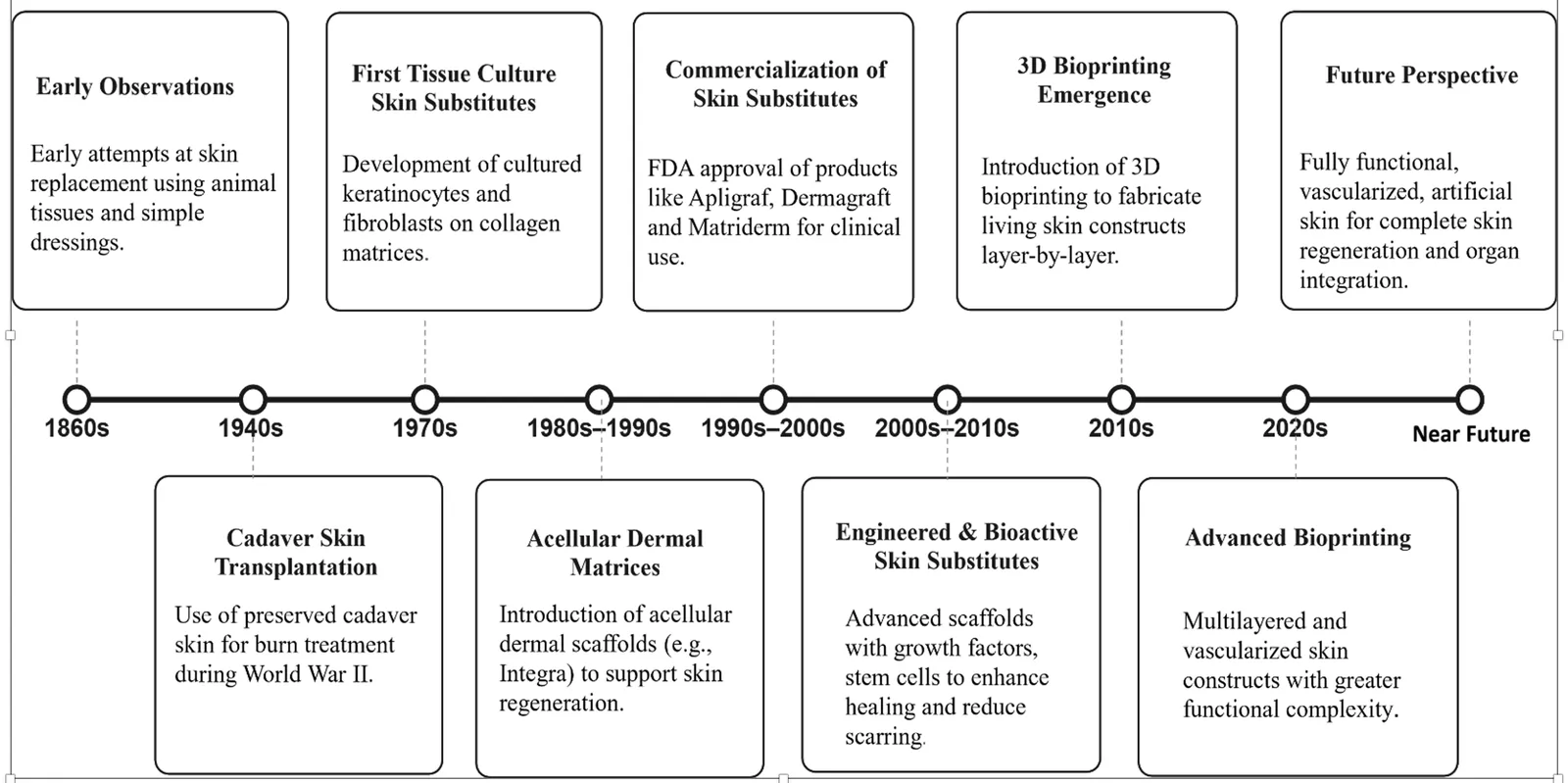
Timeline illustrating major milestones in artificial skin development Timeline illustrating major milestones in artificial skin development. Key milestones include (i) early skin grafting and chemical treatments (1800s); (ii) systemic skin transplantation techniques (1940s); (iii) Cultured epithelial autografts(1970s); (iv) Acellular dermal matrices as scaffolds for host cell infiltration (1980s – 1990s); (v) FDA approval of commercial skin substitutes (1990s-2000s); (vi) Synthetic substitutes including hydrogel and composites (2000s-2010s); (vii) 3D printing for precise skin construct fabrication (2010s); (viii) Multifunctional and microstructured skin equivalents (2010s);(ix) Development of personalized artificial skin for complex regeneration and long-term host integration (near future). Image created by the authors using Microsoft PowerPoint (Microsoft Corporation, Redmond, WA, US).

The concept of artificial skin started in the early 1860s with observations and trials to treat burn victims and trauma patients. These early endeavours established the foundational principle that external coverings could facilitate healing and reduce infection risk in open wounds [1]. However, these early attempts were limited by a lack of understanding of wound pathophysiology and the absence of suitable biomaterials, resulting in inconsistent clinical outcomes. The historical limitations underscore the importance of biomaterial development in modern tissue engineering strategies. The 1980s and 1990s introduced acellular dermal matrix (ADM) technology, exemplified by products such as Integra (Integra LifeSciences, Princeton, NJ, US), which provided a decellularized extracellular matrix derived from cadaveric or animal sources [4]. In the early twenty-first century, with the introduction of advanced engineering strategies, bioactive molecules, including growth factors (FGF, VEGF, TGF-β), bioactive peptides, and signalling molecules, were directly incorporated into skin constructs or delivered through controlled-release mechanisms. The 2010s witnessed the emergence of 3D bioprinting as a transformative technology for skin tissue engineering [5]. Due to problems like donor site scarring and tissue unavailability, researchers developed acellular and engineered skin substitutes for appendage regeneration, programmable tissue architecture, and patient-specific customization (Figure 3).

**Figure 4:**
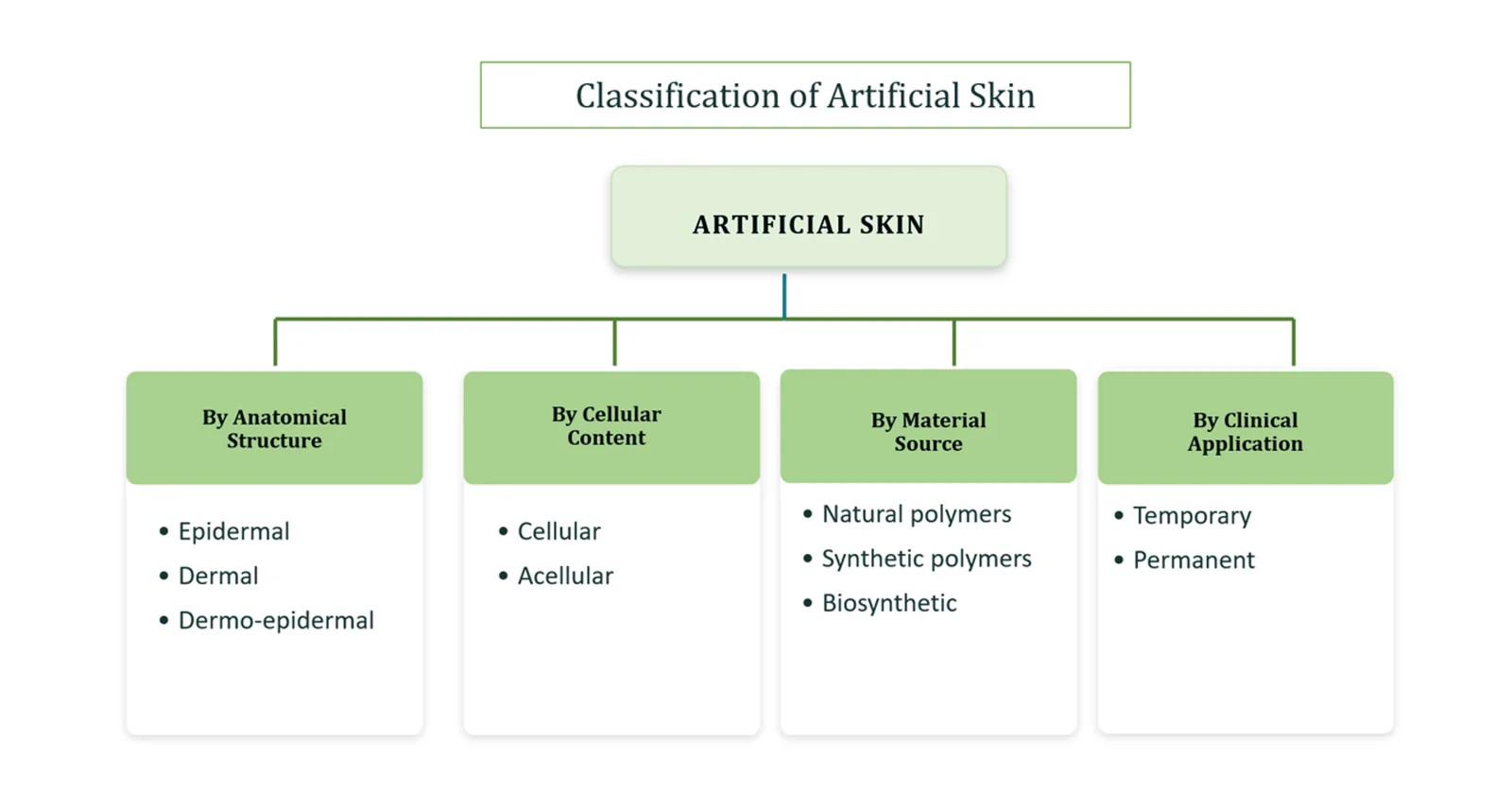
Classification of artificial skin substitutes according to anatomical structure, cellular composition, material source, and clinical application Classification of artificial skin by structure (epidermal/dermal/dermo-epidermal), cellular content (cellular/acellular), and material source (natural/synthetic/biosynthetic). This framework guides clinical selection of skin substitutes. Image created by the authors using Microsoft PowerPoint (Microsoft Corporation, Redmond, WA, US).

The production of artificial skin has been thoroughly researched to help promote wound healing and restore normal functions of the skin [17]. Clinical trials of bioengineered skin substitutes where it was found that they promote faster wound healing, lower scarring, and better outcomes for patients than traditional skin grafts [18]. Artificial skin has different types based on anatomical structures, cellular content, material source, and clinical applications (Figure 4) [2].

While there have been a number of commercially available skin substitutes, the use of such alternatives has been hindered due to various issues such as regulation, cost, and infrastructure needs for manufacturing and storage. Recent European opinions emphasize the need for harmonized regulation and a clearly defined legal framework for lab-generated autologous skin substitutes [19]. Tissue engineering in modern times has used decellularized extracellular matrix (dCEM) as a starting point to boost regenerative results. Through accurate imitation of heterogeneity in skin tissue, three-dimensional bioprinting (3D) can generate multi-layered models, which may help accurately represent the complexity of human integument [20]. While early artificial skin substitutes represented a significant advance, they were limited in their ability to replicate the complex architecture of native skin. This limitation has driven the development of 3D bioprinting technologies that offer precise control over spatial organization.

Three-dimensional bioprinting in skin tissue engineering

Three-dimensional (3D) bioprinting is an advanced additive-manufacturing technique that allows the automated and accurate placement of living cells and biological components to form complex 3D structures [21].

This technology plays a key role in regenerative medicine, as it allows the creation of personalized tissues that possess the physiological and anatomical features of natural human organs [10]. The advancement of bioprinting from industrial rapid-prototyping technology to high-resolution biological production has been fuelled by numerous breakthroughs in biomaterial synthesis and deposition technologies [22].

Historical highlights include the evolution from the conventional solid freeform fabrication process to the use of specific hydrogel systems that maintain cell viability during printing [23]. Bioprinting has been applied to overcome the limitations of traditional skin grafts, especially when dealing with full-thickness burns and diabetic ulcers [24].

**Figure 5:**
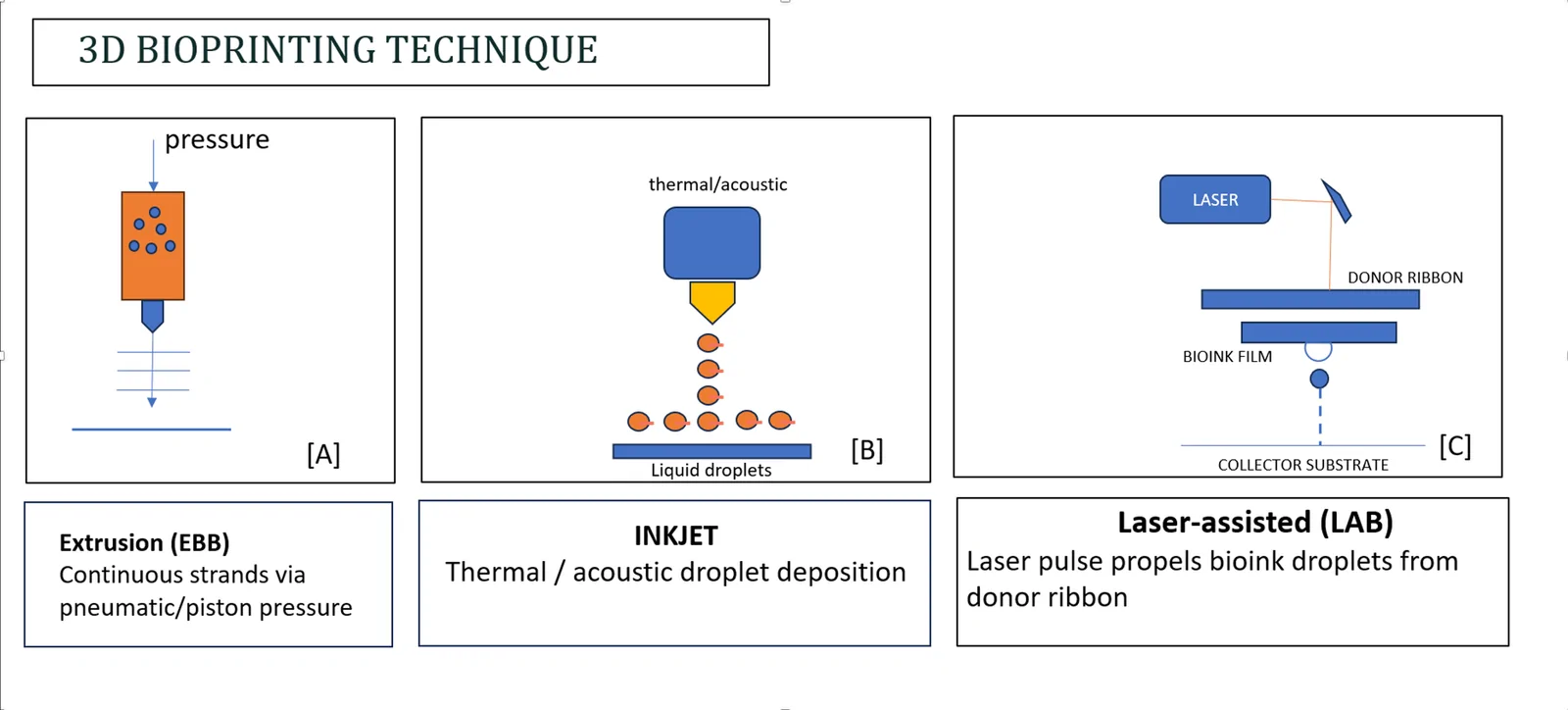
Comparison of major 3D bioprinting techniques Comparison of major 3D bioprinting techniques: (A) Extrusion-based (EBB): continuous strands via pneumatic pressure; (B) Inkjet – thermal droplets; high speed with low viscosity only; (C) Laser-assisted bioprinting (LAB): a laser pulse strikes a bioink-coated donor ribbon, generating a vapor bubble that ejects a droplet onto the collector substrate. Image created using Microsoft PowerPoint (Microsoft Corporation, Redmond, WA, US).

Different 3D bioprinting approaches have been developed for the accurate deposition of the dermis and epidermis layers in order to facilitate the reconstruction of the skin barrier and its vascular network, differing in the mechanism used to deposit the bioinks, their resolution, and cell compatibility. The main bioprinters are described in Figure 5, whereas their features are compared in Table 3 [25].

**Table 3:** Major types of bioprinting techniques

Bioprinting Technology	Description
Extrusion-based bioprinting	Uses pneumatic or mechanical force to extrude continuous bioink filaments. Suitable for high-viscosity bioinks with high cell densities [18].
Inkjet-based bioprinting	Uses thermal or piezoelectric actuators to deposit picoliter-sized droplets at high speed. Best suited for low-viscosity bioinks [19].
Laser-assisted bioprinting	Uses laser-induced forward transfer (LIFT) to print biological materials with micron-scale resolution while eliminating nozzle clogging [20].

Apart from accuracy in printing, other challenges, such as the mechanical properties of the bioprinted skin substitutes (tensile strength, elasticity, and tear resistance), still present a major challenge. New approaches to deal with the shortcomings include reinforcement with nanomaterials, crosslinking optimization, and the use of hybrid bioinks combining synthetic and natural polymers [26]. Recent advances are mainly concerned with the addition of immune and vascular components in bioprinted skin equivalents for better representation of native tissue structure [27].

Layer-by-layer deposition of bioink is the main principle of bioprinting; however, it is essential to control the "printability window" throughout to guarantee shear-thinning flow and structural recovery after deposition [25]. In this process, stabilization of shear stress is especially crucial to prevent potential damage to the cell membrane and to preserve post-printing metabolic and proliferative function [28]. However, ongoing advancements in bioprinting technology include the use of bioactive substances such as exosomes, growth factors, and extracellular matrix components to increase the potential of bioinks for regenerative purposes. These developments have been made to address the shortcomings currently faced in terms of vasculogenesis, innervation, and appendage regeneration [29]. The biomaterials and fabrication strategies developed for cutaneous repair have found increasing applicability in ophthalmology, as discussed below.

Ophthalmic applications of artificial skin and tissue-engineering technologies

The biomaterials and 3D bioprinting techniques originally developed for artificial skin and wound-healing applications share a common biological rationale with several ophthalmic tissue engineering approaches, all of which rely on biocompatible, extracellular matrix-mimicking scaffolds to support epithelial attachment, proliferation, and regeneration at a wound or defect site. This shared principle has led to the adaptation of skin-derived biomaterials across three distinct ocular applications, each addressing a different anatomical structure and clinical need. However, at present, based on the existing level of evidence, the biomaterials can be divided into established clinical applications and experimental research.

Established clinical applications

This group includes various acellular dermal allografts, which are already being used in clinical settings.

In eyelid reconstruction, acellular dermal allografts, originally developed as skin substitutes for cutaneous wound coverage, have been repurposed as posterior lamellar spacer grafts following chemical and thermal burns. Their utility in this context stems from their ability to provide structural rigidity to the eyelid while permitting host-cell repopulation, thereby reducing the risk of graft rejection compared with alternative spacer materials. Reported outcomes indicate satisfactory functional and aesthetic results, with no observed graft rejection [30].

Ongoing experimental research

This group mostly contains tissue constructs that are being extensively studied but have not entered clinical practice.

In corneal wound healing, the eggshell membrane (ESM)--a naturally fibrous, collagen-rich biomaterial studied for cutaneous regeneration--has been investigated for its ability to support corneal epithelial repair, owing to its structural similarity to native corneal stromal architecture and its favourable biocompatibility profile [31]. 

In retinal pigment epithelium (RPE) regeneration, composite hydrogels combining eggshell membrane and gellan gum, developed using formulation principles from skin tissue engineering, have been designed to support RPE monolayer formation. Here, the rationale differs from corneal and eyelid applications: rather than serving a mechanical or barrier function, these hydrogels are engineered to provide a soft, degradable substrate that replicates the biochemical properties of Bruch’s membrane, supporting RPE cell adhesion and monolayer integrity [32].

Despite these promising applications, each still faces distinct translational limitations, including long-term biomaterial stability in the corneal environment, immunogenicity profiles of allografts in eyelid reconstruction, and the mechanical durability of RPE supporting hydrogels under physiological stress, which must be addressed before broader clinical adoption.

Limitations and knowledge gaps

Skin grafts and bioengineered skin have a number of limitations due to certain methodological and physiological parameters that make them relatively ineffective. From a clinical perspective, conventional methods of skin grafting suffer from a lack of effectiveness due to donor site complications and the inability of the grafted tissue to regenerate hair follicles, sweat glands, and nerves. Bioengineered substitutes offer a promising solution to donor tissue scarcity; their use has been limited due to their high cost of production and relatively short shelf life [8]. Moreover, in chronic wounds, the abundance of proteases like elastase and MMPs destroys many growth factors applied locally [33]. In addition to these biological limitations, the clinical applications of bioprinted skin constructs suffer from other challenges, such as high production costs, non-existence of standardized manufacturing processes, difficulty in scaling up production, and the rigorous process of regulation [34]. Such constraints have resulted in the scarcity of commercially available skin products based on bioprinting technology. Lastly, the healed tissue exhibits problems in functionality, being less elastic, stiff, and unable to sense stimuli, making movement and other functions difficult and lowering the quality of life of the patients [35]. The success rate of grafts depends on many patient-specific parameters, such as smoking, nutrition, age, and the presence of comorbidities, as grafts are highly sensitive to the environment in which they are transplanted [9]. Building on these identified limitations, several emerging technologies offer potential solutions that may address current challenges in skin tissue engineering.

Future perspective and translational significance

Future prospects for artificial skin and bioprinting technologies include the use of new developments, such as 4D bioprinting, in situ robotics, smart dressings, and bio-integrated sensing technology. However, several major challenges must be overcome before the translation of such promising techniques into practice can take place, including the development of standardized manufacturing protocols, quality control measures, and scalable manufacturing procedures. Recent perspectives emphasize the need for close collaboration among researchers, doctors, regulatory agencies, and other stakeholders [36]. The technology of 4D bioprinting employs stimuli-responsive materials, including shape-memory polymers that respond to changing pH or the temperature of wounds and hence adaptively regenerate tissues. Smart dressings that can contain biosensors can monitor the condition of the wound in terms of parameters like pH, glucose concentration, and temperature to provide controlled delivery of drugs to the site of the wound. Bio-integrated sensing technology will allow the monitoring of the local wound microenvironment in real time through feedback [37]. There is also the development of an in situ robotics bioprinting system that can deposit skin substitutes in a wounded area during surgery [38]. In summary, while the field has made substantial progress, the translation of these technologies into clinical practice requires continued research and development.

## Conclusions

The review traces the progression from conventional skin grafting to 3D printing, highlighting the increasing relevance of cutaneous biomaterials and fabrication strategies to ophthalmology. Through layer-by-layer deposition, 3D bioprinting has the potential to overcome key graft limitations and may offer a patient-specific route to functional tissue. Preclinical in vivo studies have reported encouraging outcomes, including accelerated re-epithelialization, organized collagen deposition, and progressive vascularization, suggesting that bioprinting can translate rationally designed bioinks into structurally competent skin substitutes. However, these findings derive largely from heterogeneous animal studies with limited human clinical evidence. Significant challenges remain, including high production costs, lack of standardization, and rigorous regulatory pathways. Future work must prioritize vascularized, multi-layer constructs alongside robust clinical validation and regulatory alignment to facilitate translation.
